# Functional Reorganizations of Brain Network in Prelingually Deaf Adolescents

**DOI:** 10.1155/2016/9849087

**Published:** 2015-12-27

**Authors:** Wenjing Li, Jianhong Li, Jieqiong Wang, Peng Zhou, Zhenchang Wang, Junfang Xian, Huiguang He

**Affiliations:** ^1^College of Electronic Information and Control Engineering, Beijing University of Technology, Beijing 100124, China; ^2^State Key Laboratory of Management and Control for Complex Systems, Institute of Automation, Chinese Academy of Sciences, Beijing 100190, China; ^3^Beijing Key Laboratory of Computational Intelligence and Intelligent System, Beijing 100124, China; ^4^Department of Radiology, Beijing Tongren Hospital, Capital Medical University, Beijing 100730, China; ^5^Department of Radiology, Beijing Friendship Hospital, Capital Medical University, Beijing 100050, China

## Abstract

Previous neuroimaging studies suggested structural or functional brain reorganizations occurred in prelingually deaf subjects. However, little is known about the reorganizations of brain network architectures in prelingually deaf adolescents. The present study aims to investigate alterations of whole-brain functional network using resting-state fMRI and graph theory analysis. We recruited 16 prelingually deaf adolescents (10~18 years) and 16 normal controls matched in age and gender. Brain networks were constructed from mean time courses of 90 regions. Widely distributed network was observed in deaf subjects, with increased connectivity between the limbic system and regions involved in visual and language processing, suggesting reinforcement of the processing for the visual and verbal information in deaf adolescents. Decreased connectivity was detected between the visual regions and language regions possibly due to inferior reading or speaking skills in deaf subjects. Using graph theory analysis, we demonstrated small-worldness property did not change in prelingually deaf adolescents relative to normal controls. However, compared with healthy adolescents, eight regions involved in visual, language, and auditory processing were identified as hubs only present in prelingually deaf adolescents. These findings revealed reorganization of brain functional networks occurred in prelingually deaf adolescents to adapt to deficient auditory input.

## 1. Introduction

Prelingual deafness is the hearing loss that occurs at birth or before the onset of speech. Due to the deprivation of auditory inputs, brain plasticity has been reported by numerous neuroimaging studies. “Cross-modal plasticity” has been suggested in deaf subjects, which is represented by the phenomenon that the auditory cortex can be activated when deaf subjects perform various tasks, such as speech [1, 2] and visual tasks [3, 4]. Many morphological studies did not find structural changes in the primary auditory cortex [5–8], indicating the atrophy of the auditory cortex due to hearing loss could be compensated by the use of this cortex for other stimuli. However, brain regions involved in visual and speech processing have been found to change in deaf subjects [7, 8], indicating that the sensory systems for vision and speech might participate in the tasks which are supposed to activate the auditory system in healthy controls. Therefore, we speculated that the cooperative manner for different brain regions would be altered in prelingually deaf subjects when dealing with a complex task.

The human brain is a highly complex system with synchronized neural activity from different brain regions. The concept of “connectome” was first proposed by Sporns et al. in 2005 [9], which represents the human brain as an interconnected network. Functional brain network refers to a pattern of statistical dependencies between distinct brain regions. The network architecture identified by the analysis of functional connectivity could be an effective pattern to present the cooperative manner for brain regions, and it reflects the potential anatomical connections between brain regions as well. To further quantitatively measure the brain network, network topological properties could be evaluated at both global and regional levels using graph theory, which becomes a promising tool for analyzing brain networks in recent years [10–21]. Small-worldness, a concept that originated from social network, quantifies the effectiveness of information transferring within networks and has been successfully used to characterize brain networks [14–16]. Besides, nodal topological parameters based on graph theory analysis are used to illustrate the properties for nodes which are defined as brain regions, identifying the role of specific regions in transferring information. It has been demonstrated that these measures of brain network are sensitive to aging [17, 18] as well as various neuropsychiatric diseases, such as schizophrenia [16, 19] and Alzheimer's disease [20, 21]. Therefore, investigation of brain network properties provides a new insight into brain reorganization and is critical to understand the working mechanism of brains with hearing loss.

Recently, a few studies have emerged to investigate the structural or functional connectivity between brain regions in deaf subjects. Kim et al. [22] examined morphological brain network in deaf adults using tissue density on MRI and analyzed the network properties using graph theory and network filtration. They found altered morphological network in prelingually deaf adults compared to normal controls but not in postlingually deaf adults, concluding that auditory experience could affect the morphology of brain networks in deaf adults. Besides, Li et al. [23] employed resting-state fMRI to investigate the effect of deafness on the intra- and interregional synchronization of different parts of superior temporal sulcus and revealed that the intrinsic function of these different parts are distinctly impacted by deafness. However, these previous studies focused on alterations in grown adult brains of deaf subjects but not developing brain. Adolescence is an important transitional stage for brain development, in which brain's structure and function are in developmental changes to accommodate to external environment. Therefore, adolescents with prelingual deafness are supposed to go through a different pattern of brain reorganization to adapt to the silent world relative to normal adolescents, which is supported by one of our previous studies [24] using structural MRI to investigate grey matter connectivity within and between auditory, language, and visual systems in deaf adolescents. To the best of our knowledge, the whole-brain functional network and its properties have not been investigated in prelingually deaf adolescents yet.

We hypothesized the architectures or properties of brain network would change in prelingually deaf adolescents. In the present study, we used resting-state fMRI and graph theory analysis to investigate alterations of whole-brain functional networks in prelingually deaf adolescents. Functional brain network was constructed based on mean time series extracted from 90 brain regions. Functional connectivity patterns were studied in deaf and control groups, respectively, and compared between two groups. The global network property of small-worldness was evaluated in each group and group differences were investigated as well. Besides, nodal topological parameters were calculated and brain hubs were identified for deaf and control groups, respectively. Nodal properties were finally compared in all the hubs between prelingually deaf adolescents and normal controls.

## 2. Materials and Methods

### 2.1. Subjects

In this study, we recruited 16 prelingually deaf adolescents and 16 normal controls with matched age (*p* = 0.815) and gender (Chi-Squared *p* = 1.0), which were the same as the data used in our previous studies [7, 8, 24]. All prelingually deaf adolescents suffered from severe sensorineural hearing loss after the birth and had a mean pure tone audiometry average air conduction threshold greater than 90 dB of hearing loss for the better ear and no single frequency better than 45 dB of hearing loss between 500 and 4000 Hz. All deaf subjects wore hearing aids and learnt Chinese sign language. Furthermore, all subjects had neither history of central nervous system disease nor significant head trauma. All subjects underwent an identical resting-state fMRI paradigm. The data from one prelingually deaf adolescent and one normal control were discarded due to excessive head movement during scanning. Therefore, 15 deaf subjects (age 14.32 ± 2.24 years, 10~18 years; 8 males) and 15 normal controls (age 14.81 ± 2.07 years, 10~18 years; 8 males) remained, and these two groups were still matched in age (*p* = 0.769) and gender (Chi-Squared *p* = 1.0). Detailed biographical information of all subjects is shown in Table 1. This project was approved by the Committee at Beijing Tongren Hospital, and all subjects and their parents gave written informed consent before inclusion.

### 2.2. Data Acquisition

Resting-state fMRI data were collected from all subjects on a 3-Tesla MR imaging scanner (GE Medical System, Milwaukee, WI, USA) with an eight-channel phased-array head coil. To acquire resting-state fMRI data, all subjects lay on their backs and were instructed explicitly to keep their eyes closed and not to think of anything in particular. Foam padding was used to limit head motion and reduce scanner noises. The scanning parameters were as follows: repetition time (TR) = 2000 ms, echo time (TE) = 30 ms, field of view (FOV) = 24 × 24 cm^2^, matrix size = 64 × 64, slice thickness = 5 mm, and flip angle = 90°, yielding 28 axial slices with in-plane resolution of 3.75 × 3.75 cm^2^. The fMRI scanning lasted 400 s and 200 volumes were obtained for each subject.

Furthermore, high-resolution T1-weighted structural brain images were collected for structural reference using a 3D SPGR sequence (TR = 9 ms, TE = 3.5 ms, inversion time (TI) = 450 ms, FOV = 24 × 24 cm^2^, matrix size = 256 × 256, slice thickness = 1 mm, and flip angle = 13°, yielding 196 sagittal slices with in-plane resolution of 0.9375 × 0.9375 cm^2^).

### 2.3. Data Preprocessing

The preprocessing steps were performed using the Statistical Parametric Mapping (SPM8, http://www.fil.ion.ucl.ac.uk/spm, Wellcome Department of Cognitive Neurology, London, UK, 2008) on a Matlab 7.9 platform (MathWorks, Natick, MA, USA). The first 10 volumes of the resting-state fMRI data were discarded because of the initial transient effects and the adaption of the subjects to the environment. The remaining 190 time points were corrected for within-scan acquisition time differences between slices and realigned to the first image for head movement correction, resulting in 3 translational and 3 rotational parameters. The subjects with excessive head movement (translation > 2 mm and rotation degree > 3°) were thus excluded. Subsequently, the functional scans were spatially normalized to a standard space (Montreal Neurological Institute, MNI) using the normalization parameters from T1 image with high resolution to structural T1 template in MNI space and resampled to 3 × 3 × 3 mm^3^. The images were then smoothed with a 6 mm full width at half maximum (FWHM) isotropic Gaussian kernel. After that, the preprocessed images were linearly drifted and passed through a band-pass filter (0.01–0.08 Hz) to remove the effects of low frequency drift and physiological high frequency respiratory and cardiac noise. We regressed out six parameters of head movement as well as mean signals of the whole brain, white matter, and cerebral spinal fluid.

### 2.4. Network Construction

The functional images were parcellated into 90 brain regions (45 regions for each hemisphere) based on Automated Anatomical Labelling (AAL) template (see Table 2). To construct the whole-brain network, time courses were first extracted from all voxels within each ROI and averaged. Then, Pearson's correlation coefficient was calculated between pairs of regions throughout the whole brain, resulting in a 90 × 90 correlation matrix. Larger correlation coefficients indicate more synchronized time courses between the corresponding pairs of regions, implying stronger functional connectivity between these two regions.

### 2.5. Graph Theory Analysis

Brain network properties were analyzed using graph theory based on the correlation matrix, in which 90 ROIs and their connections were considered as nodes and edges, respectively. The correlation matrix was binarized by a given threshold, resulting in a sparse matrix. We adjusted the threshold and obtained a series of connectivity matrices with different sparsities. Using graph theory analysis, we characterized the global topological properties of brain functional networks using the parameters of small-worldness (*σ*), which was defined as [25]

(1) $$\begin{array}{c} \sigma =\frac{\gamma }{\lambda }=\frac{C_{p}^{\text{real}}/C_{p}^{\text{rand}}}{L_{p}^{\text{real}}/L_{p}^{\text{rand}}}, \end{array}$$

where *γ* and *λ* are normalized clustering coefficient and normalized path length, respectively. The normalized clustering coefficient is the ratio of clustering coefficient of a real network *C*_*p*_^real^ to that of a random network *C*_*p*_^rand^, and the normalized path length is defined as the ratio of characteristic path length of a real network *L*_*p*_^real^ to that of a random network *L*_*p*_^rand^.

Here, clustering coefficient *C*_*p*_ is a measure of the degree to which nodes in a network cluster together and is defined as

(2) $$\begin{array}{c} C_{p}=\frac{1}{n}\overset{n}{\underset{i=1}{\sum }}\frac{{2E}_{i}}{D_{\text{nod}}\left(i\right)\left(D_{\text{nod}}\left(i\right)-1\right)}, \end{array}$$

where *D*_nod_(*i*) is the degree of the node *i*, which is defined in the following part of this section, *E*_*i*_ is the total number of edges connecting the node *i* with the nodes which are the nearest neighbors to the node *i*, and *N* is the number of nodes in the network.

Characteristic path length *L*_*p*_ is the average shortest path length between two nodes over all pairs of nodes and is defined as

(3) $$\begin{array}{c} L_{p}=\frac{\sum_{i}\sum_{j}L_{ij}}{n\left(n-1\right)}, \end{array}$$

where *n* is the number of nodes in the network and *L*_*ij*_ is the shortest path length between nodes *i* and *j*.

A network is said to have the property of small-worldness if it satisfies *λ* ≈ 1 and *γ* ≫ 1 or *σ* = *γ*/*λ* ≫ 1.

It has been demonstrated that the sparsity of 12% is close to an optimal threshold at which most informative network edges are retained and disconnections between nodes are rare [26, 27]. Therefore, we chose 12% as the sparsity threshold for analysis of nodal topological properties, including betweenness centrality, nodal degree, and nodal efficiency, which are defined as follows.

*(1 ) Betweenness Centrality*. Betweenness centrality *C*_*b*_ is a measure of the number of times that a node is along the shortest path between other two nodes, reflecting the importance of the individual node in the overall network structure. It is computed as follows:

(4) $$\begin{array}{c} C_{b}=\underset{s\neq i\neq t\in N}{\sum }\frac{\delta_{st}\left(i\right)}{\delta_{st}}, \end{array}$$

where *N* is the set of all nodes in the network, *δ*_*st*_ is the total number of shortest paths from node *s* to node *t*, and *δ*_*st*_(*i*) is the number of the paths that pass through the node *i*.

*(2 ) Nodal Degree*. Nodal degree of the node *i* (*D*_nod_(*i*)) in a network is the number of connections linking the node with all other nodes and is defined as

(5) $$\begin{array}{c} D_{\text{nod}}\left(\;i\;\right)=\underset{j\in N}{\sum }a_{ij}, \end{array}$$

where *N* is the set of all nodes in the network and *a*_*ij*_ is the connection status between node *i* and node *j* (*a*_*ij*_ = 1 when the connection exists, and *a*_*ij*_ = 0 otherwise).

*(3 ) Nodal Efficiency*. Nodal efficiency of the node *i* (*E*_nod_(*i*)) is the mean of the inverse shortest path length from node *i* to all other nodes and is defined as

(6) $$\begin{array}{c} E_{\text{nod}}\left(\;i\;\right)=\frac{1}{n}\underset{j\neq i}{\sum }\frac{1}{L_{ij}}, \end{array}$$

where *n* is the number of nodes in the network and *L*_*ij*_ is the shortest path length between nodes *i* and *j*.

### 2.6. Statistical Analysis

#### 2.6.1. Analysis of Whole-Brain Functional Connectivity

When constructing whole-brain network using resting-state fMRI, the correlation matrix characterized the brain functional connectivity pattern for each subject. To identify significant connectivity pattern in each group, one-sample *t*-test was performed to find significantly positive correlation in deaf group and normal controls, respectively, indicating significant connections between pairs of regions throughout the whole brain. To compare differences of connectivity patterns between groups, Fisher's *R*-to-*Z* transformation was first performed and then two-sample *t*-test was conducted on the correlation matrices between prelingually deaf adolescents and normal controls. These statistical results were corrected for multiple comparisons using partial Bonferroni correction which considers the correlation between the dependent variables. This correction method was implemented using the Simple Interactive Statistical Analysis Bonferroni tool (http://www.quantitativeskills.com/sisa/calculations/bonfer.htm), which optimally balanced Type-I and Type-II errors. When performing one-sample *t*-test, the mean correlation coefficient between all variables in the matrix was 0.2236 in control group and 0.2274 in deaf group, leading to an equivalent corrected *p* of 7.94*e* − 5 for control group and 8.63*e* − 5 for deaf group (number of tests = 90 *∗* 89/2 = 4005), respectively. For analysis of group differences, the mean correlation coefficient between pairs of variables for all subjects was 0.1624, resulting in an equivalent corrected *p* of 4.83*e* − 5.

#### 2.6.2. Analysis of Network Topological Properties

To present the properties of brain functional network for each group, the property of small-worldness was analyzed at different sparsities (from 10% to 45%) for prelingually deaf adolescents and normal controls, respectively. Then, two-sample *t*-test was performed between two groups to figure out the group differences.

As described above, we chose 12% as the sparsity threshold for analysis of nodal topological properties. First, the mean betweenness centrality, mean nodal degree, and mean nodal efficiency were calculated for each node in deaf and normal groups, respectively. Then, we ranked them among all the nodes with the highest score (90) for the highest betweenness centrality, nodal degree, and nodal efficiency, respectively, and the scores for each measure were summed up for each node. The top 20% nodes (18 out of 90 nodes) with high scores were identified as hub nodes in each group. The three nodal topological parameters were then compared between two groups for the hubs using two-sample *t*-test and the results were corrected for multiple comparisons using partial Bonferroni correction as well.

## 3. Results

### 3.1. Whole-Brain Functional Connectivity Pattern

**Figure 1:**
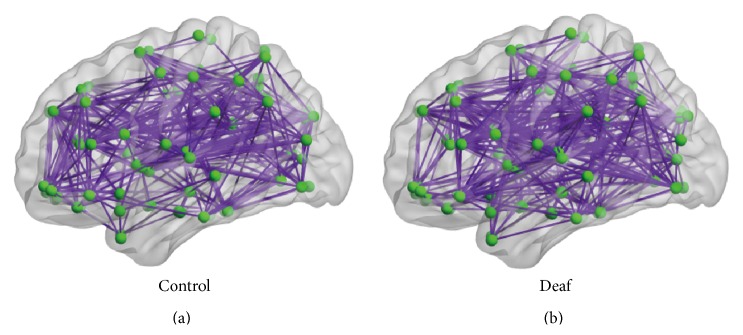
3D views of functional connectivity patterns in normal controls (a) and prelingually deaf adolescents (b).

Using one-sample *t*-test, significant functional connectivity was detected for both prelingually deaf adolescents and normal controls after partial Bonferroni correction (as shown in Figure 1). The 3D view in Figure 1 was visualized with the BrainNet Viewer (http://www.nitrc.org/projects/bnv) [28]. The figure shows functional connectivity was widely distributed throughout the whole brain in both groups.

**Figure 2:**
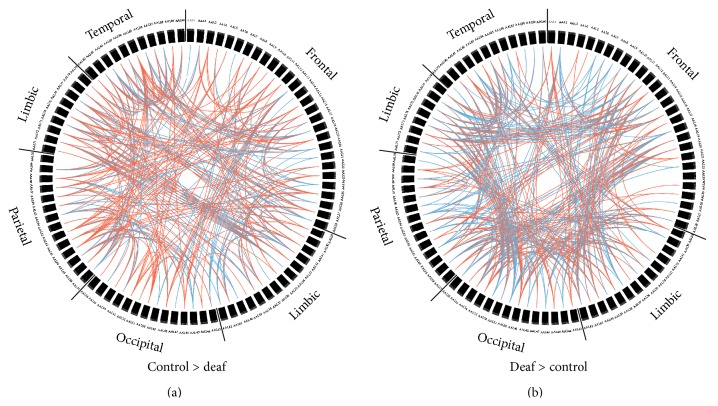
Group differences of brain functional connectivity between prelingually deaf adolescents and normal controls (uncorrected, *p* < 0.05). (a) represents stronger functional connectivity in normal controls, while (b) shows stronger connectivity in prelingually deaf subjects. The red color indicates unilateral connections, and the blue color indicates bilateral connections.

**Figure 3:**
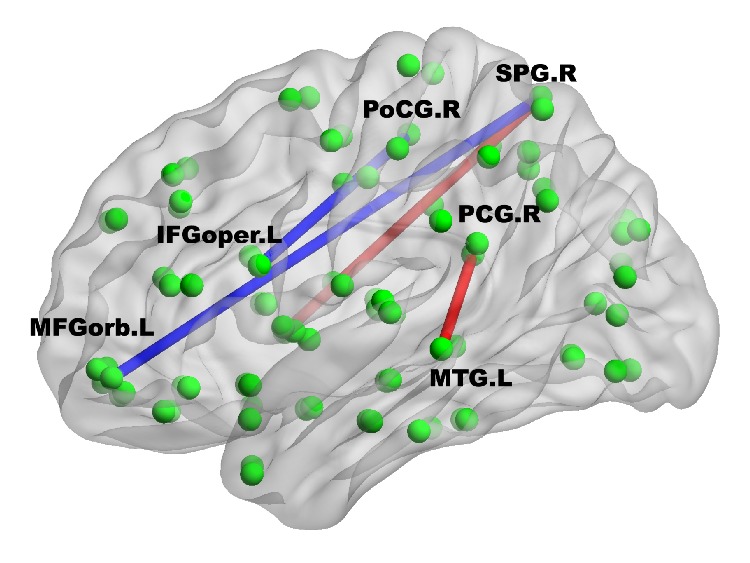
The 3D view for group differences after correction for multiple comparisons. While the warm color represents stronger connectivity in prelingually deaf adolescents compared to normal controls, the cold color suggests weaker connectivity.

To identify group differences of functional connectivity throughout the whole brain, connectivity matrices were compared between two groups.  Figure 2 shows the group differences before correction for multiple comparisons. From the figure, we can see that prelingually deaf adolescents show weaker connectivity between the regions within the temporal lobe, but stronger connectivity between regions in the occipital lobe and regions in the limbic system and temporal lobe. After partial Bonferroni correction, prelingually deaf adolescents showed significantly increased functional connectivity between the right superior gyrus (SPG) and right insula (INS) and between the left middle temporal gyrus (MTG) and right posterior cingulate gyrus (PCG) when compared with normal controls (see Figure 3). Besides, significantly decreased connectivity was found in deaf group between the right SPG and left middle frontal gyrus (orbital part, MFGorb) and between the right postcentral gyrus (PoCG) and left inferior frontal gyrus (opercular part, IFGoper).

### 3.2. Properties of Brain Functional Network

**Figure 4:**
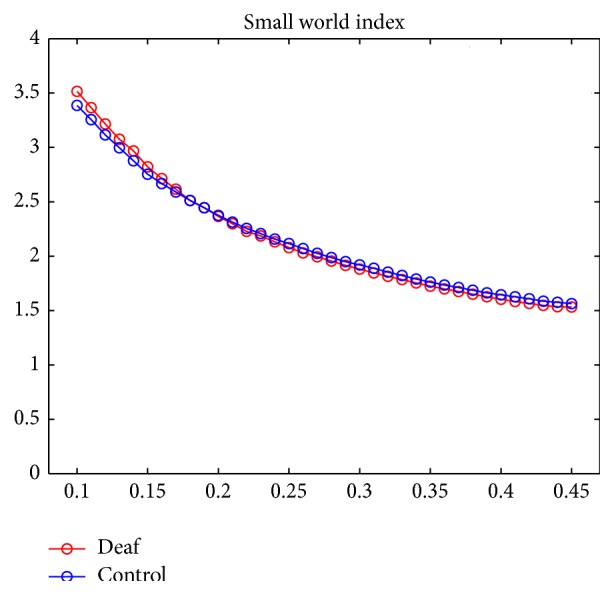
Small world index *σ* across sparsities from 10% to 45% with an interval of 1% in prelingually deaf adolescents (red color) and normal controls (blue color). Both of the two groups present the property of small-worldness since *σ* ≫ 1.

Small-worldness *σ* of brain functional network was measured at different sparsity (from 10% to 45% with an interval of 1%) in deaf group and control group, respectively. As shown in Figure 4, we found *σ* ≫ 1 across all the sparsities in both groups, indicating that the network has the property of small-worldness. We did not find significant differences of the small world index between prelingually deaf adolescents and normal controls.

**Figure 5:**
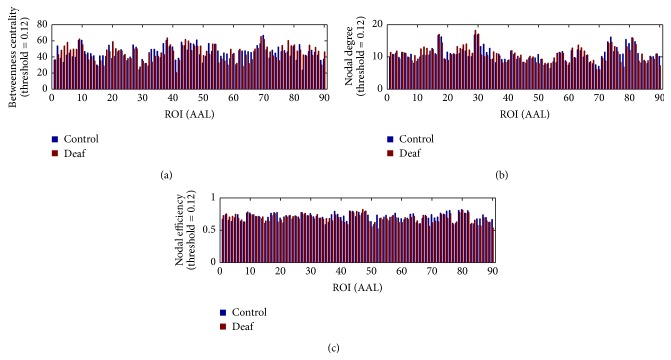
Bar plots for the average value for regional topological properties of 90 ROIs, including betweenness centrality (a), nodal degree (b), and nodal efficiency (c), in control and deaf groups. The blue color indicates the control group, and the red color represents the deaf group.

Besides the global network property, the regional topological properties, including betweenness centrality, nodal degree, and nodal efficiency, were calculated in each ROI.  Figure 5 shows the mean values for these regional properties in control and deaf groups.

### 3.3. Hubs of Brain Network

**Figure 6:**
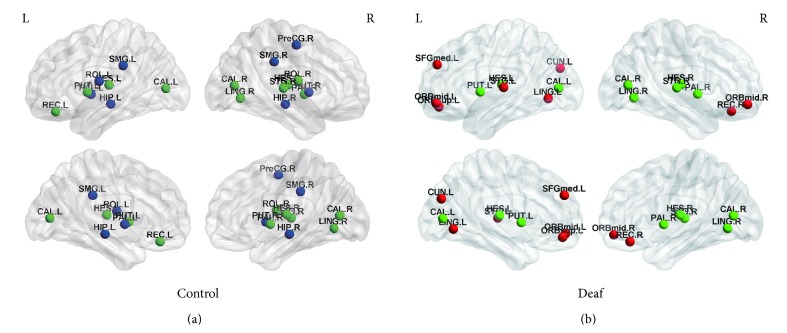
Hubs for brain functional networks in control (a) and deaf (b) groups. The hubs identified in both groups are shown in green color. While the hubs for the control group only are present with blue color, the hubs for the deaf group only are shown in red color.

The hub in the network is supposed to have relatively high betweenness centrality, nodal degree, and nodal efficiency. Based on the method described in Section 2, we identified 18 regions out of 90 ROIs as the hubs in each group at the sparsity of 12% since a network with this sparsity tends to retain the edges with most information and ensure disconnection is rare. As shown in Figure 6 and Table 3, ten brain regions, including the right rolandic operculum (ROL), left gyrus rectus (REG), bilateral calcarine fissure (CAL), right lingual gyrus (LING), left putamen (PUT), right pallidum (PAL), bilateral Heschl gyrus (HES), and right superior temporal gyrus (STG), were the hubs in both deaf and control groups. Besides, the middle and superior frontal gyri and cuneus (CUN) were hubs in deaf group but not in control group, while the right precentral gyrus (PreCG), bilateral hippocampus (HIPP), and supramarginal gyrus (SMG) were hubs only in control group.

### 3.4. Group Differences of Topological Parameters for Hubs

There were a total of 26 brain regions identified as hubs either in deaf or in control groups. The nodal topological parameters, including betweenness centrality, nodal degree, and nodal efficiency, were compared for these nodes between prelingually deaf adolescents and normal controls. Significant differences did not survive after correction for multiple comparisons. However, we found trends for group differences, as listed in Table 3. Betweenness centrality is higher in the left superior frontal gyrus (orbital part, SFGorb) (*p* = 0.0204, not corrected) and is lower in the left ROL (*p* = 0.0480), SMG (*p* = 0.0251), and PAL (*p* = 0.0221) in deaf group relative to control group. Besides, prelingually deaf adolescents have higher nodal degree in the left middle frontal gyrus (orbital part, MFGorb) (*p* = 0.0484) and left superior frontal gyrus (medial part, SFGmed) (*p* = 0.0302) and lower nodal efficiency in the left ROL (*p* = 0.0367), left hippocampus (*p* = 0.0442), and left pallidum (*p* = 0.0015).

## 4. Discussions

The present study employed resting-state fMRI and graph theory analysis to investigate changes of brain functional network in prelingually deaf adolescents. Functional connectivity was widely distributed in both deaf and control groups, and significant differences were found for connections between the right SPG and right insula, the left MTG and right PCG, the right SPG and left MFGorb, and the right PoCG and left IFGoper. The whole-brain network has the property of small-worldness in both prelingually deaf adolescents and normal controls. Ten regions were identified as the hubs in both groups. While the middle and superior frontal gyri and CUN were the hubs that emerged only in deaf group, the right PreCG, bilateral HIPP, and SMG were hubs only in control group. These findings provide new evidence for brain reorganization in prelingually deaf adolescents and could help to understand the working mechanism in the period of brain development after hearing loss.

In prelingually deaf adolescents, functional connectivity was widely distributed as observed in normal controls but differed in the connectivity pattern. Significantly increased connectivity was detected between the right SPG and right insula and between the left MTG and right PCG. The insula and PCG are in limbic system, which are thought to play an important role in recruiting relevant brain regions for sensory information processing [29]. The SPG is supposed to receive considerable visual input and is involved in visual-spatial relations [30]. Besides, the MTG has been reported to be one of the critical nodes in the brain's language network, which can access word meaning while reading [31]. Therefore, our findings of increased connectivity between these regions suggest that the limbic system in prelingually deaf adolescents could reinforce the processing of the visual and verbal information due to the deprivation of auditory sensory processing. Additionally, we found decreased functional connectivity between the right SPG and left MFGorb and between the right PoCG and left IFGoper. Evidence suggests that the left MFG is the specific brain region for Chinese reading [32], and the left IFG is extremely important for language comprehension and production [33]. The decreased connectivity might be caused by the inferior skills of reading or speaking observed in most of the deaf subjects [34, 35].

Although it is not significant, we found a trend of significance for group differences in functional connectivity pattern. Connectivity between the regions within the temporal lobe was found to be weaker in prelingually deaf adolescents, indicating that the auditory cortex are less activated to auditory stimuli. In contrast, stronger connectivity was observed between regions in the occipital lobe and regions in the temporal lobe, which were identified as primary visual and auditory cortices, respectively. This result is consistent with one of our findings in the previous study [23], which detected significantly stronger structural connections between visual and auditory systems in prelingually deaf adolescents.

Using graph theory analysis, whole-brain network holds the property of small-worldness in both prelingually deaf adolescents and normal controls. The small world index was almost the same across all the sparsities from 10% to 45% (with an interval of 1%) between the two groups (see Figure 3). This result suggests that the effectiveness of information transferring of the brain network was not affected even without auditory inputs in prelingually deaf adolescents.

In the brain network, hubs are regions playing an important role in facilitating communication among parallel, distributed brain networks [36]. Eighteen hubs were identified in each group. In control group, the hubs included the right PreCG, bilateral ROL, left REG, bilateral HIPP, bilateral CAL, right LING, bilateral PUT, bilateral PAL, bilateral HES, and right STG. Compared with healthy adolescents, middle and superior frontal gyri, left LING, left CUN, left STG, and right REG were hubs only observed in prelingually deaf adolescents. Evidences show that the STG is the primary auditory cortex and is involved in phonological processing for speech perception and production as well [37, 38]. Although not activated by auditory stimuli, auditory regions could be activated by other stimuli [1–4] as stated in Section 1. Therefore, auditory regions might become a brain hub connecting with other brain regions. In the hubs of deaf subjects, the LING and CUN are associated with visual processing [39, 40]. To adapt to deficient auditory input, prelingually deaf adolescents could rely critically on vision to interact with the external environment. Our findings of the hubs in LING and CUN suggest the improvement of visual performance in deaf adolescents, supporting cross-modal changes in visual processing. Of note, deaf subjects recruited in the present study learn Chinese Sign Language (CSL). It has been demonstrated that the middle and superior frontal gyri can be activated during observing and imitating CSL [41]. We speculated that the hubs of MFG and SFG only observed in prelingually deaf subjects might be caused by the use of CSL. All the above changes indicate brain reorganizations after hearing loss in prelingually deaf adolescents.

There are still some limitations in the present study. First, the sample size is relatively small. We recruited 16 prelingually deaf adolescents and 16 normal controls, and 15 subjects remained for each group after removing subjects with excessive head movement. More subjects will be scanned in further studies. Second, the effects of clinical parameters, such as the information for the use of sign language and hearing aids, were not analyzed in the present study, which will be taken into consideration in future.

## 5. Conclusions

The present study employed resting-state fMRI and graph theory to investigate alterations of brain functional network in prelingually deaf adolescents compared to normal controls. Functional connectivity was widely distributed in prelingually deaf adolescents. Increased connectivity was significantly found between the limbic system and visual and language-related regions, suggesting reinforcement of the processing for the visual and verbal information in deaf adolescents. Decreased connectivity was detected between the visual regions and language regions possibly due to inferior reading or speaking skills in deaf subjects. Graph theory analysis revealed that brain functional network holds small-worldness in both prelingually deaf adolescents and normal controls. Compared with healthy adolescents, middle and superior frontal gyri, left LING, left CUN, left STG, and right REG were hubs only observed in prelingually deaf adolescents. These hubs were involved in visual, language, and auditory processing, reflecting brain reorganization to adapt to deficient auditory input.

## Figures and Tables

**Table 1:** Biographical information of two groups.

	Normal controls	Deaf adolescents
Number	15	15
Male/female	8/7	8/7
Mean age (years)	14.81	14.32
Standard deviation (SD) age (years)	2.07	2.24
Age range (years)	10~18	10~18

**Table 2:** Regions of interest (ROI) defined in AAL template.

Region name	Abbr.	Labels	
		L	R
Precentral gyrus	PreCG	1	2
Superior frontal gyrus, dorsolateral	SFGdor	3	4
Superior frontal gyrus, orbital	SFGorb	5	6
Middle frontal gyrus	MFG	7	8
Middle frontal gyrus, orbital	MFGorb	9	10
Inferior frontal gyrus, opercular	IFGoper	11	12
Inferior frontal gyrus, triangular	IFGtri	13	14
Inferior frontal gyrus, orbital	IFGorb	15	16
Rolandic operculum	ROL	17	18
Supplementary motor area	SMA	19	20
Olfactory cortex	OLF	21	22
Superior frontal gyrus, medial	SFGmed	23	24
Superior frontal gyrus, medial orbital	SFGmorb	25	26
Gyrus rectus	REG	27	28
Insula	INS	29	30
Anterior cingulate gyrus	ACG	31	32
Median cingulate gyrus	MCG	33	34
Posterior cingulate gyrus	PCG	35	36
Hippocampus	HIP	37	38
Parahippocampal gyrus	PHIP	39	40
Amygdala	AMYG	41	42
Calcarine fissure	CAL	43	44
Cuneus	CUN	45	46
Lingual gyrus	LING	47	48
Superior occipital gyrus	SOG	49	50
Middle occipital gyrus	MOG	51	52
Inferior occipital gyrus	IOG	53	54
Fusiform gyrus	FG	55	56
Postcentral gyrus	PoCG	57	58
Superior parietal gyrus	SPG	59	60
Inferior parietal gyrus	IPG	61	62
Supramarginal gyrus	SMG	63	64
Angular gyrus	ANG	65	66
Precuneus	PCNU	67	68
Paracentral lobule	PCL	69	70
Caudate	CAU	71	72
Putamen	PUT	73	74
Pallidum	PAL	75	76
Thalamus	THA	77	78
Heschl gyrus	HES	79	80
Superior temporal gyrus	STG	81	82
Superior temporal gyrus, temporal pole	STGp	83	84
Middle temporal gyrus	MTG	85	86
Middle temporal gyrus, temporal pole	MTGp	87	88
Inferior temporal gyrus	ITG	89	90

**Table 3:** Group differences of nodal topological parameters in hubs (not corrected).

Brain region	Hubs		BC		ND		NE	
	Deaf	Control	D > C	D < C	D > C	D < C	D > C	D < C
PreCG.R	nonhub	hub	ns	ns	ns	ns	ns	Ns
SFGorb.L	hub	nonhub	0.0204	ns	ns	ns	ns	Ns
MFGorb.L	hub	nonhub	ns	ns	0.0484	ns	ns	Ns
MFGorb.R	hub	nonhub	ns	ns	ns	ns	ns	Ns
ROL.L	nonhub	hub	ns	0.0480	ns	ns	ns	0.0367
ROL.R	hub	hub	ns	ns	ns	ns	ns	Ns
SFGmed.L	hub	nonhub	ns	ns	0.0302	ns	ns	Ns
REG.L	hub	hub	ns	ns	ns	ns	ns	Ns
REG.R	hub	nonhub	ns	ns	ns	ns	ns	Ns
HIP.L	nonhub	hub	ns	ns	ns	ns	ns	0.0442
HIP.R	nonhub	hub	ns	ns	ns	ns	ns	Ns
CAL.L	hub	hub	ns	ns	ns	ns	ns	Ns
CAL.R	hub	hub	ns	ns	ns	ns	ns	Ns
LING.L	hub	nonhub	ns	ns	ns	ns	ns	Ns
LING.R	hub	hub	ns	ns	ns	ns	ns	Ns
SMG.L	nonhub	hub	ns	0.0251	ns	ns	ns	Ns
SMG.R	nonhub	hub	ns	ns	ns	ns	ns	Ns
CUN.L	hub	nonhub	ns	ns	ns	ns	ns	Ns
PUT.L	hub	hub	ns	ns	ns	ns	ns	Ns
PUT.R	nonhub	hub	ns	ns	ns	ns	ns	Ns
PAL.L	nonhub	hub	ns	0.0221	ns	ns	ns	0.0015
PAL.R	hub	hub	ns	ns	ns	ns	ns	Ns
HES.L	hub	hub	ns	ns	ns	ns	ns	Ns
HES.R	hub	hub	ns	ns	ns	ns	ns	Ns
STG.L	hub	nonhub	ns	ns	ns	Ns	ns	Ns
STG.R	hub	hub	ns	ns	ns	Ns	ns	Ns

D > C: deaf > control; D < C: deaf < control; ns: not significant.
